# Time-dependent image changes after ethanol injection into the pancreas: an experimental study using a porcine model

**DOI:** 10.3332/ecancer.2016.663

**Published:** 2016-08-15

**Authors:** Kazuyuki Matsumoto, Hironari Kato, Koichiro Tsutsumi, Soichiro Fushimi, Masaya Iwamuro, Shinsuke Oda, Sho Mizukawa, Yutaka Akimoto, Daisuke Uchida, Takeshi Tomoda, Naoki Yamamoto, Shigeru Horiguchi, Hiroyuki Okada

**Affiliations:** 1 Department of Gastroenterology and Hepatology, Okayama University Graduate School of Medicine, Dentistry, and Pharmaceutical Science, 2-5-1 Shikata-cho, Okayama 700-8558, Japan; 2 Department of Pathology, Okayama University Graduate School of Medicine, Dentistry, and Pharmaceutical Science, 2-5-1 Shikata-cho, Okayama 700-8558, Japan

**Keywords:** ethanol injection, pancreas, porcine model, ultrasound, magnetic resonance imaging

## Abstract

**Background:**

Ethanol, a commonly available agent, has been used to successfully ablate cystic and solid lesions in the pancreas. The aim of this study is to investigate the effects of an ethanol injection into the porcine pancreas and observe the time-dependent image changes in the pancreatic parenchyma.

**Methods:**

Pure ethanol was injected into the pancreatic tail using a 25-gauge EUS needle with direct ultrasound guidance under celiotomy: 1 mL and 2 mL were injected, respectively. The abdomen was closed after the injection. MRI was performed before the procedure, immediately after, and on postoperative day (POD) seven. Blood samples were taken before the procedure and on PODs one, three, five, and seven. The pigs were euthanised on POD seven.

**Results:**

Immediately after the injection, linear high signal areas in the pancreatic tail on T2 and rounded speckled high signal areas on DWI images were detected in both animals, measuring 35 × 32 mm in the 1 mL injected pig and 42 × 38mm in the 2 mL injected pig. After POD seven, rounded high signal areas were noted on T2 images, measuring 22 × 18 mm and 36 × 28 mm respectively. On POD one, the 1 mL injected animal had a 53% elevation in serum amylase while the 2 mL injected animal had a 66% elevation. Histologically, cystic and necrotic changes in the parenchyma were observed, measuring 23 × 22 mm and 40 × 35 mm respectively.

**Conclusions:**

Our results, which are limited to normal pancreas, suggested that a 1 mL injection caused localised changes within the pancreas while a 2 mL injection induced more widespread changes beyond the pancreas. The effective area of ethanol was widespread immediately after injection, and then the area was reduced with cystic and necrosis changes.

## Introduction

Endoscopic ultrasound (EUS) and EUS-guided fine-needle aspiration (EUS-FNA) are recognised as useful and safe modalities for providing high-resolution images and tissue samples for diagnosis of pancreatic neoplasms [1–2]. Recently, the EUS-FNA technique was modified to provide an injection modality to deliver therapeutic agents for several pancreatic diseases [3–4].

Ethanol is a particularly attractive agent. It is inexpensive, readily available, and has the potential to rapidly ablate tissue. Its purported mechanisms of action are cell lysis and protein denaturation [5]. Ethanol ablation of the normal porcine pancreas was reported using EUS guidance [6–8]. Furthermore, treatments by EUS-guided ethanol injection for pancreatic cystic tumours, pancreatic neuroendocrine tumours, pancreatic adenocarcinoma, and metastatic lesions in humans have been reported [9–15]. However, the details of image changes in the area of ablation after the injection of ethanol have not been shown.

Magnetic resonance imaging (MRI) is a technology that can detect the location and local chemical environment of protons in water molecules. MRI with diffusion weighted imaging (DWI) sequences and apparent diffusion coefficient (ADC) maps is widely accepted as a very sensitive way to detect acute changes. The reduced diffusion is thought to be related to cytotoxic oedema and shrinkage of the extracellular space [16, 17]. MRI could evaluate the condition of injured tissues.

The purpose of this study was to investigate the area of ablation in the pancreas using US-guided ethanol injection under celiotomy and time-dependent image changes of pancreatic parenchyma after the ethanol injection by MRI.

## Methods

**Figure 1.**
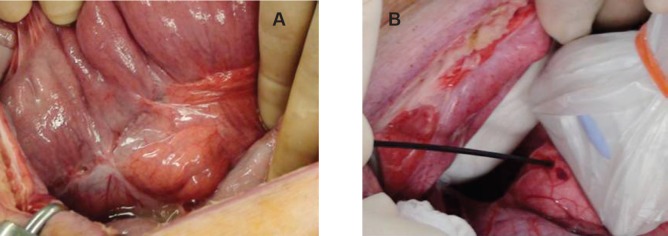
Exposed pancreatic tail by celiotomy and selected injection area under US. a: The tail of the pancreas was exposed by celiotomy. b: A 25-gauge EUS needle was inserted into the thickest portion of the pancreatic tail under direct US guidance.

Two female Yorkshire pigs (average weight 35 kg) were used for this study. This experimental protocol was approved by the Institutional Review Board of the Intervention Technical Centre (IVTeC Co., Ltd., Tokyo, Japan) for the Use of Laboratory Animals in the Medical Device Development Centre (MEDDEC, Kobe, Japan) before the initiation of the study. The pigs were anesthetised with intramuscular ketamine and xylazine along with isoflurane that was delivered via endotracheal tube. After adequate anesthesia was achieved, the tail of the pancreas was exposed by celiotomy. We observed the pancreas directly using US (Aplio 300; Toshiba Medical Systems Corp., Tokyo, Japan) and identified the thickest portion of the pancreatic tail. A 25-gauge EUS needle (Expect; Boston Scientific, Tokyo, Japan) was inserted into the thickest portion of the pancreatic tail under US guidance (Figure 1a, b). The needle was primed with 100% ethanol solution (Mylan; Pfizer, Tokyo, Japan) before the injection. The first animal received 1.0 mL ethanol which was slowly injected into the pancreatic parenchyma. In the second animal 2.0 mL ethanol was administered. Hyperechoic bubbles created by the ethanol were continuously visualised during the injection (Video 1). After finishing the ethanol injection, the abdomen was closed.

MRI (Excite XI Twin Speed 1.5T; GE Healthcare, Tokyo, Japan) of the abdomen was performed before the procedure, immediately after, and on POD seven. Images of T1- and T2-weighted, fat-sat T1 and T2-weighted, magnetic resonance cholangiopancreatography (MRCP), DWI, and ADC were taken after administration of rocuronium. The slice thicknesses of T1, T2, and DWI were 4.5 mm, and MRCP images were processed to create reformatted images of 0.6-mm thick. Blood samples were taken before the procedure and on PODs one, three, five, and seven. Serum concentrations of amylase (AMY) and lactate dehydrogenase (LDH) and a complete blood count (CBC) were assessed. Animals were monitored for lethargy, severe distention, and vomiting, with observation until POD seven. For the first two PODs, each animal was administered 500 mg/day of cefazolin sodium. Animals had access to regular solid food during this time.

After animals were given anesthesia on POD seven, a laparotomy was performed after which the animals were euthanised while still under anesthesia. The gross appearance of the pancreas and surrounding structures was recorded. The pancreas was harvested, examined, palpated, and sectioned to identify any gross abnormalities. Tissue samples were preserved in 10% formalin for histological analysis with haematoxylin and eosin (H.E.) staining.

## Results

Each animal tolerated the US-guided ethanol injection under the celiotomy without clinical manifestations of pancreatitis or distress. Activity and dietary intake in both animals were normal over the seven-day observation period.

### MRI findings of the pancreas (Figure 2–5)

**Figure 2.**
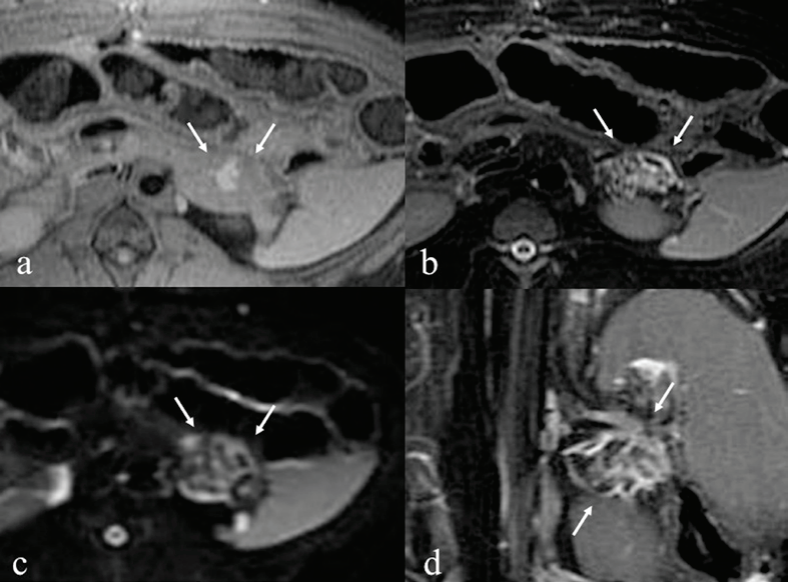
MRI immediately after injection with 1.0 mL ethanol. a: T1-weighted image shows small high signal areas measuring 12 x 7 mm in the pancreatic tail. b: T2-weighted image shows linear high signal areas measuring 35 x 32 mm. c: DWI image shows rounded speckles high signal areas. d: T2 coronal image.

**Figure 4.**
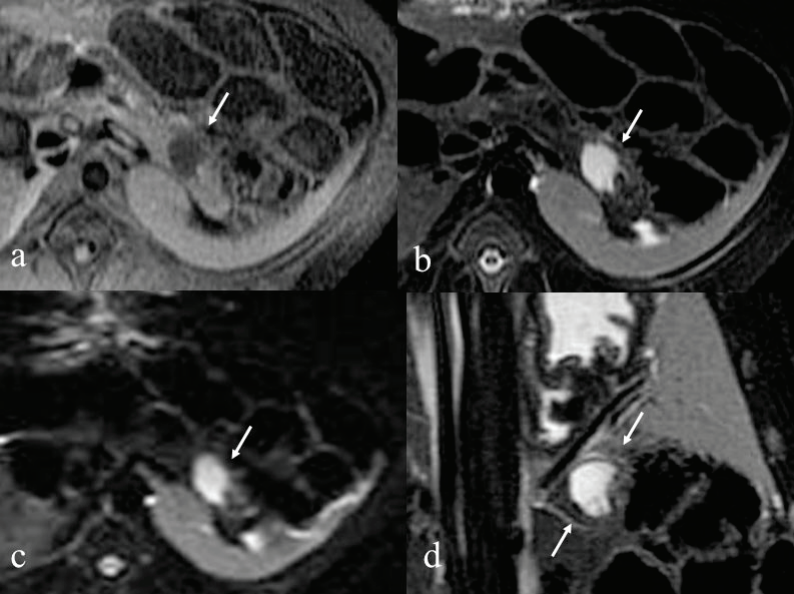
After seven days MRI of 1.0 mL injected pig. a: T1-weighted image shows low signal areas in the tail of pancreas. b: T2-weighted image shows rounded high signal areas measuring 22 x 18 mm. c: DWI image shows rounded high signal areas similar to T2 image. d: T2 coronal image.

Immediately after the injection of ethanol, linear high signal areas in the pancreatic tail were noted in both animals on T2-weighted images; high signal areas were 35 × 32 mm in the animal administered 1.0 mL ethanol and 42 × 38 mm in the animal administered 2.0 mL ethanol. On the other hand, small high signal areas were noted in both animals on T1-weighted images. On the DWI, speckled high signal areas were detected in both animals (Figure 2a-d, 3a-d). On POD seven, a round high signal area in the animal administered 1.0 mL ethanol and an irregular high signal area in the animal administered 2.0 mL ethanol were noted on T2-weighted images, measuring 22 × 18 mm and 36 × 28 mm, respectively. On T1-weighted images, a low signal area was noted in the animal administered 1.0 mL ethanol with approximately the same measurement as on T2-weighted images. On the other hand, an irregular low signal area and part of a high signal area were noted in the animal administered 2.0 mL ethanol on T2-weighted images. On the DWI, high signal areas were clearly detected in both animals, and they were same as shown in T1 and T2-weighted images (Figure 4a-d, 5a-d). It was difficult to identify the main pancreatic duct in both animals on the MRCP image. There were no findings of acute pancreatitis by MRI.

### Biochemical evaluation (Figure 6)

**Figure 6.**
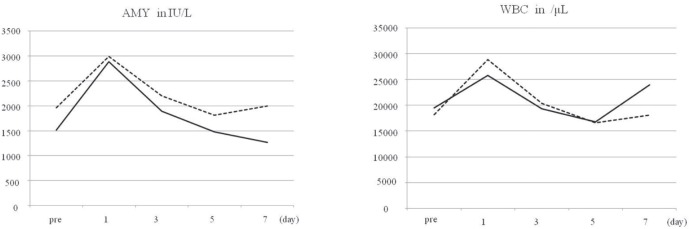
Biochemical changes after ethanol injection. Serum levels of a: amylase (AMY); b: white blood cells (WBC) plotted against time. Solid line shows data for 1.0 mL-injected pig and dotted line shows data for 2.0-mL injected pig.

On POD one, the animal receiving 1.0 mL of ethanol had a 53% elevation (1518–2890 U/L) in serum AMY while the animal receiving 2.0 mL of ethanol had a 67% elevation (1962–2933 U/L) of AMY (Figure 6a). Elevations in the WBC of 76% (19500–25800/uL) and 63% (18200–28900/uL) were noted in animals receiving 1.0 mL and 2.0 mL of ethanol respectively (Figure 6b). However, both parameters returned to baseline on POD three. Haemoglobin was slightly decreased in both animals on POD seven (1.0 mL animal: 12.6–10.6 g/dL, 2.0 mL animal: 14.1–13.3 g/dL), but values were not decreased sufficiently to result in clinical problems. The serum LDH value was elevated on POD seven in the 1.0 mL-injected animal (724–932 U/L), but no such elevation was noted at any evaluation point in the 2.0 mL-injected animal (489–488 U/L).

### Gross pathology and histology (Figures 7–8)

**Figure 7.**
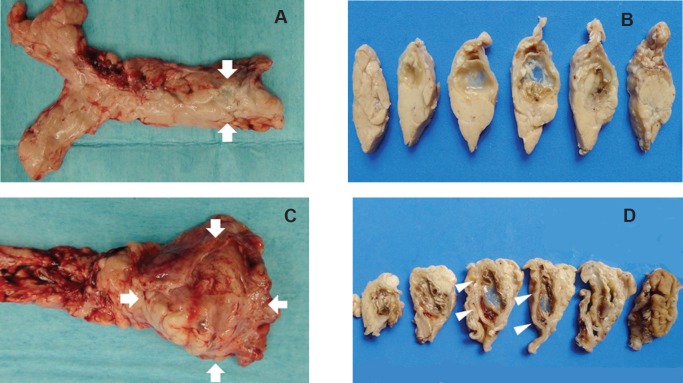
Macroscopic view of the extirpated pancreas (a, b: 1.0 mL-injected pig, c, d: 2.0 mL-injected pig). a: The injected area was slightly pale and swollen (arrow). b: Cross section shows cystic change at the injected area. c: Large swelling at the injected area (arrow). d: The cross section shows a cystic change of the injected area and adhesion between the pancreatic tail and small intestine (arrow-head).

Macroscopically, tissues around the injection site were pale and swollen. Especially, in the 2.0 mL-injected animal, the injection site of the pancreatic tail was greatly swollen and had adhered to the small intestine (Figure 7a–d). Cross-section showed a cystic change at the injected area, and the content of it was serous clear liquid. Loupe images showed cystic and necrotic changes in the parenchyma, measuring 23 × 22 mm in the 1.0 mL-injected pig and 40 × 35 mm in the 2.0-ml injected pig. Histological examination of the pancreas revealed wide areas of coagulative necrosis and cystic changes with saponification of parenchyma at the site of injection, but no evidence of generalised pancreatitis. Inflammatory cell infiltration was seen around the sites of necrosis, and an extremely severe inflammatory cell infiltration to the small intestine was observed in the 2.0 mL-injected animal (Figure 8a-f). The pig injected with 1.0 mL 100% ethanol showed tissue necrosis and cystic changes within the pancreatic parenchyma. On the other hand, the pig injected with 2.0 mL ethanol developed a larger area of necrosis and a cystic change beyond the pancreatic parenchyma. Table 1 shows the image changes of pancreatic parenchyma evaluated by each MRI method and actual parenchymal changes measured by histology.

## Discussion

A previous study of EUS-guided ethanol injection into the normal porcine pancreas showed that 50% ethanol (N = 4, 0.5 mL) caused localised and self-limited changes with focal areas (2–6 mm) of inflammation, necrosis, and fibrosis at the injection site. On the other hand, the pigs injected with 98% ethanol (N = Four; 1.0 mL was one; 0.5 mL were three) developed more widespread tissue changes with large areas (8–30 mm) of inflammation, fibrosis, and necrosis, and local complications of pancreatitis: acute fluid collection in one case and inflammatory colonic stricture in the other case [6]. Another porcine study of EUS-guided 100% ethanol injection (N = Two, 2.0 mL) caused pancreatic tissue necrotic changes of 15 × 8 mm and 13 × 7 mm respectively [8]. A similar study of 100% ethanol (N = One, 2.0 mL) showed pancreatic tissue necrotic changes measuring 23 × 16 mm [7]. In our study, the pig injected with 1.0 mL 100% ethanol using direct US guidance under laparotomy showed tissue necrosis and cystic changes (23 × 22 mm) within the pancreatic parenchyma. Conversely, the pig injected with 2.0 mL ethanol developed a larger area of necrosis and cystic change (40 × 35 mm) beyond the pancreatic parenchyma. As the result of ethanol leakage surrounding the pancreas, severe inflammatory cell infiltration into the small intestine was observed. Our results suggested that a 1.0 mL injection of 100% ethanol caused localised changes with an approximately 20 mm ablation area within the pancreas, whereas the 2.0 mL injection induced more widespread changes with about a 40 mm area of ablation beyond the pancreas. This might be because of the difference in body weight between pigs (other study: 45–50 kg; our study: 35 kg). Therefore, the pancreas in our study might have been smaller than that in other studies, making it easier for injected ethanol to spread beyond the pancreatic parenchyma. As a result, the ablated areas were extended.

MRI is a technology that uses nonionising radiofrequency radiation inside a strong magnetic field to detect the location and local chemical environment of protons in water molecules. MRI with DWI sequences and ADC maps is widely accepted as a very sensitive way to detect acute changes, especially in the field of cerebral infarctions [16]. The reduced diffusion is thought to be related to cytotoxic oedema and shrinkage of the extracellular space [17]. The mechanism of ethanol injection therapy is that ethanol induces cellular dehydration and protein denaturation followed by coagulation necrosis [5]. In our experimental study, speckled high signal areas on the DWI map immediately after injection probably means a shrinkage of the extracellular space by cellular dehydration effect of pure ethanol. Small high signal areas on T1-weighted images might mean a little bleedings of injected area. Linear high signal areas on T2-weighted images might show ethanol spread to pancreatic parenchyma. After seven days, the bleeding was naturally absorbed and the ethanol ablation area produced a low signal on T1-weighted and a high signal on T2-weighted images, namely the component was suspected to be close to the water. The areas of ablation shown by T1 and T2-weighted images and DWI were almost in the same range as the histologically detected areas. The detailed changes after ethanol ablation could be observed by using MRI. In addition, intravenous injection of contrast agents is not necessary for MRI scans including for DWI, so there is no risk of allergic reactions to contrast medium.

Clinically, ethanol ablation is commonly used to ablate hepatocellular carcinomas [18]. Premalignant lesions of the liver are also sensitive to ethanol ablation [19]. Ethanol injections are being expanded to treatment of other organs such as the thyroid gland [20], the prostate gland [21] and the gastrointestinal stromal tumours [22]. In pancreatic diseases, EUS-guided ethanol lavage therapy for pancreatic cystic lesions was reported with effective results [9]. The complications of ethanol lavage of cysts rarely included extravasation of ethanol outside of the cystic cavity [9]. Recently EUS-guided ethanol injection for patients with a small pancreatic neuroendocrine tumour, who could not be treated with medical therapy, or refused surgery, or were poor surgical candidates, produced effective results with rarely any complications [10–14]. Moreover, EUS-guided ethanol ablation of a tumour combined with celiac plexus neurolysis in patients with pancreatic adenocarcinoma were reported with effective pain control and prolongation of overall survival(OS) [15].

There are a few limitations to this study. Firstly, the present study investigated only on a few animals, and therefore a more detailed assessments of the safety and efficacy of this procedure is necessary. Secondly, the study findings are applicable only to normal pancreas. Ethanol ablation is usually used for malignant or premalignant lesions. Therefore, the present findings might be different from actual clinical findings. Third, although the changes in the images (before the procedure, immediate after, and POD seven) were observed by MRI, no information was available after a week.

## Conclusion

In conclusion, our results which are limited to normal pancreas, suggest that a 1.0 mL injection of 100% ethanol caused localised changes within the pancreas whereas a 2.0 mL injection induced more widespread changes beyond the pancreas. Image changes in the pancreatic parenchyma after the ethanol injection could be observed by using T1 and T2-weighted and DWI imaging. It was found that the effective areas of ethanol were widespread immediately after injection, and then these areas reduced with cystic and necrosis changes. MRI without contrast agents could correctly evaluate areas of ethanol ablation.

## Conflict of interest

The authors declare that they have no conflicts of interest associated with this study.

## Author contributions

Matsumoto K, Kato H and Fushimi S conceived and designed the research and wrote the paper; Tsutsumi K, Iwamuro M, Oda S, and Mizukawa S, analysed and interpreted the data; Akimoto Y, Uchida D, Tomoda T, Yamamoto N, and Horiguchi S did critical revision of the article for important intellectual content; and Okada H gave final approval of this article.

## Institutional review board statement

The study was reviewed and approved by the Institutional Review Board of the Intervention Technical Centre (IVTeC Co., Ltd., Tokyo, Japan) for the Use of Laboratory Animals in the Medical Device Development Centre (MEDDEC, Kobe, Japan).

## Institutional animal care and use committee statement

All experimental manipulations were undertaken in accordance with the Guide for the Care and Use of Laboratory Animals (National Institutes of Health), and the study was approved by the Intervention Technical Centre (IVTeC Co., Ltd., Tokyo, Japan) for the Use of Laboratory Animals in the Medical Device Development Centre (MEDDEC, Kobe, Japan).

## Figures and Tables

**Figure 3.**
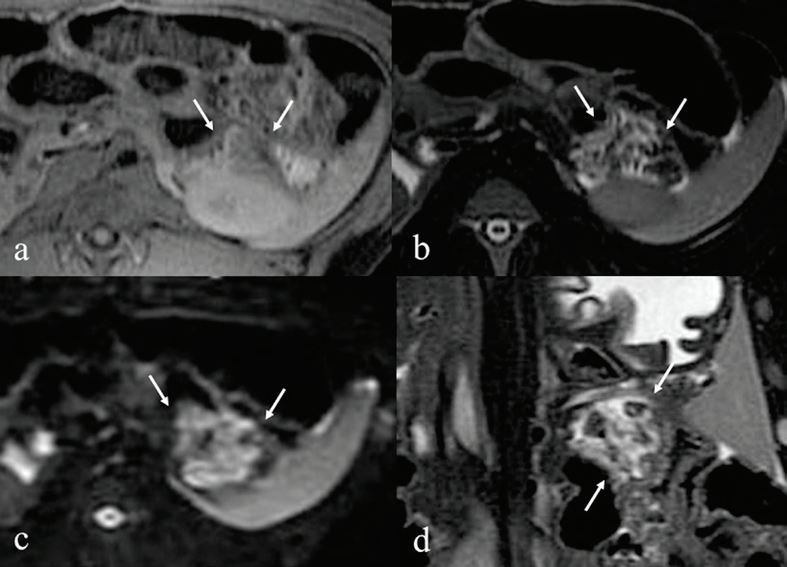
MRI immediately after injection with 2.0 mL ethanol. a: T1-weighted image shows small high and low signal areas measuring 20 x 15 mm in the tail of pancreas. b: T2-weighted image showed linear high signal areas, measuring 42 x 38 mm. c: DWI image showed rounded speckles high signal areas. d: T2 coronal image.

**Figure 5.**
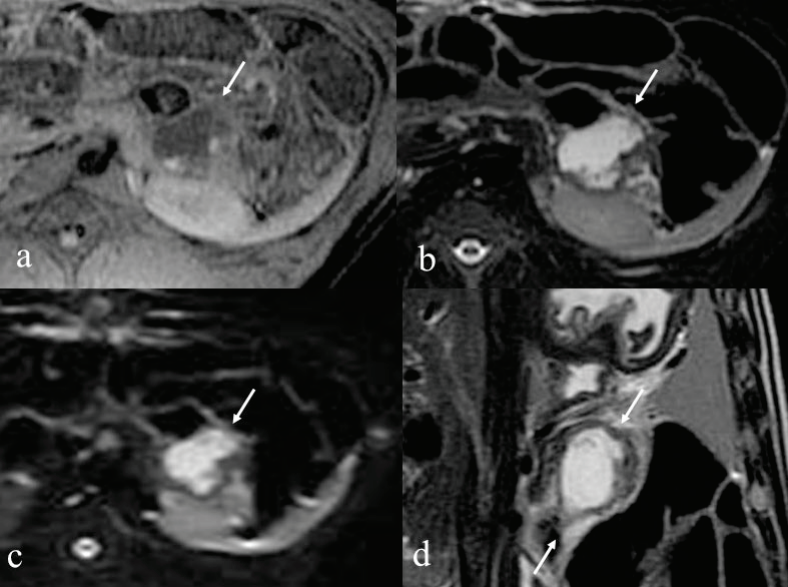
After seven days MRI of 2.0 mL injected pig. a: T1-weighted image shows low signal area and part of a high signal areas in the tail of pancreas. b: T2-weighted image reveals irregular high signal areas measuring 36 x 28 mm. c: DWI image reveals irregular high signal areas similar to T2 image. d: T2 coronal image.

**Figure 8.**
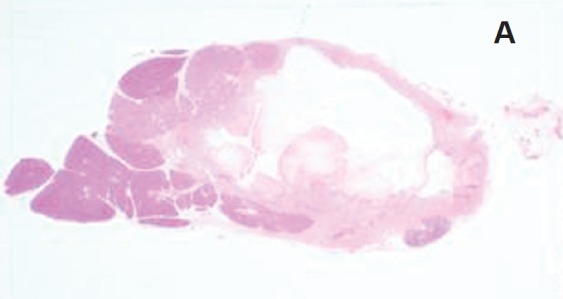
Histological findings of 1.0 mL- (a-c) and 2.0 mL- (d-f) injected animals. a: Loupe image reveals 23 x 22 mm cystic change in the pancreas parenchyma.

**Figure 8. b, c:**
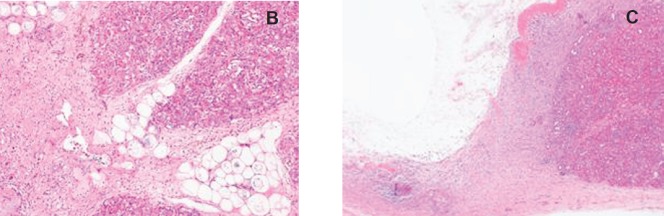
Photomicrographs of cystic and necrotic areas of parenchyma around the injection site (H.E.). Fat necrosis in pancreatic interlobular tissue was barely accompanied by inflammatory cell infiltration. Inflammatory cell infiltration is evident around the site of injection.

**Figure 8. d:**
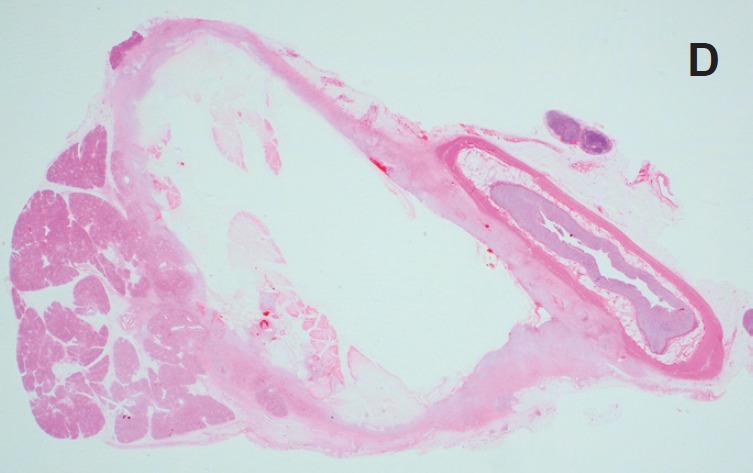
Loupe image showed 40 x 35 mm cystic change in the pancreatic parenchyma and adhesion to the small intestine.

**Figure 8. e, f:**
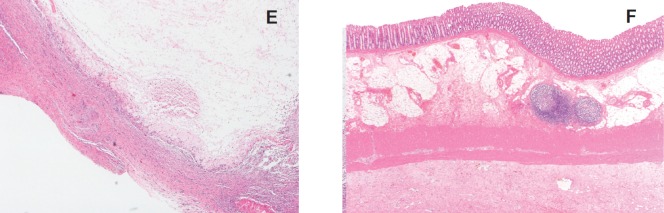
Necrosis and inflammation extended beyond the pancreatic parenchyma.

**Video 1.**
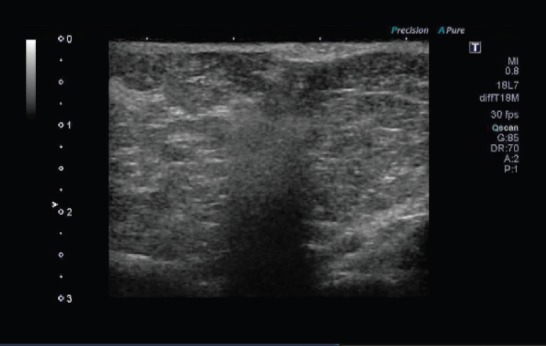
We observed the pancreas directly using US and could identify the thickest portion of the pancreatic tail. A 25-gauge EUS needle was inserted into the thickest portion of the pancreatic tail under US guidance. The needle was primed with 100% ethanol solution before the injection and 1.0 mL ethanol was slowly injected into the pancreatic parenchyma. Hyperechoic bubbles created by ethanol were continuously visualised during injection.

**Table 1.** Image changes of pancreatic parenchyma evaluated by each method of MRI and the actual parenchyma changes measured by histology.

	T1-weighted (immediately after / POD 7)	T2-weighted (immediately after / POD 7)	DWI (immediately after / POD 7)	Histology (POD seven)
1.0 mL injected pig, mm	12 × 7 / 22 × 18	35 × 32 / 22 × 18	40 × 38 / 22 × 18	23 × 22
2.0 mL injected pig, mm	20 × 15 / 36 × 28	42 × 38 / 36 × 28	50 × 42 / 36 × 28	40 × 35

POD; postoperative day, DWI; diffusion weighted imaging

## References

[1] Chang KJ (1997). The clinical utility of endoscopic ultrasound-guided fine-needle aspiration in the diagnosis and staging of pancreatic carcinoma. Gastrointest Endosc.

[2] Gress FG (1997). Endoscopic ultrasound-guided fine-needle aspiration biopsy using linear array and radial scanning endosonography. Gastrointest Endosc.

[3] Krinsky ML (2000). EUS-guided investigational therapy for pancreatic cancer. Gastrointest Endosc.

[4] Gress F (2001). Endoscopic ultrasound-guided celiac plexus block for managing abdominal pain associated with chronic pancreatitis: a prospective single center experience. Am J Gastroenterol.

[5] Gelczer RK (1998). Complications of percutaneous ethanol ablation. J Ultrasound Med.

[6] Aslanian H (2005). EUS-guided ethanol injection of normal porcine pancreas: a pilot study. Gastrointest Endosc.

[7] Matthes K (2007). Concentration-dependent ablation of pancreatic tissue by EUS-guided ethanol injection. Gastrointest Endosc.

[8] Matsumoto K (2008). Endoscopic ultrsound-guided ethanol injection in the pancreas in a porcine model: a preliminary study. J Gastroenterol Hepatol.

[9] Gan SI (2005). Ethanol lavage of pancreatic cystic lesions: initial pilot study. Gastrointest Endosc.

[10] Jurgensen C (2006). EUS-guided alcohol ablation of an insulinoma. Gastrointest Endosc.

[11] Levy MJ (2012). US-guided ethanol ablation of insulinomas: a new treatment option. Gastrointest Endosc.

[12] Zhang WY (2013). Endoscopic ultrasound-guided ethanol ablation therapy for tumors. World J Gastroenterol.

[13] Park do H (2015). Endoscopic ultrasonography-guided ethanol ablation for small pancreatic neuroendocrine tumors: results of a pilot study. Clin Endsc.

[14] Paik WH (2016). Safety and efficacy of EUS-guided ethanol ablation for treating small solid pancreatic neoplasm. Medicine (Baltimore).

[15] Facciorusso A (2016). Echoendoscopic ethanol ablation of tumor combined with celiac plexus neurolysis in patients with pancreatic adenocarcinoma. J Gastroenterol Hepatol.

[16] Benveniste H (1992). Mechanism of detection of acute cerebral ischemia in rats by diffusion-weighted magnetic resonance microscopy. Stroke.

[17] Lovblad KO (1998). Clinical experience with diffusion-weighted MR in patients with acute stroke. AJNR Am J Neuroradiol.

[18] Livraghi T (2001). Percutaneous ethanol injection in the treatment of hepatocellular carcinoma in cirrhosis. Hepatogastroenterology.

[19] Lencioni R (1994). Percutaneous ethanol injection therapy of adenomatous hyperplastic nodules in cirrhotic liver disease. Acta Radiol.

[20] Kim JH (2003). Efficacy of sonographically guided percutaneous ethanol injection for treatment of thyroid cysts versus solid thyroid nodules. Am J Roentgenol.

[21] Chiang PH (2003). Pilot study of transperineal injection of dehydrated ethanol in the treatment of prostatic obstruction. Urology.

[22] Gunter E (2003). EUS-guided ethanol injection for treatment of a GI stromal tumor. Gastrointest. Endosc.

